# A disposable biosensor based on immobilization of laccase with silica spheres on the MWCNTs-doped screen-printed electrode

**DOI:** 10.1186/1752-153X-6-103

**Published:** 2012-09-17

**Authors:** Yuanting Li, Li Zhang, Meng Li, Zhigang Pan, Dawei Li

**Affiliations:** 1 Key Laboratory for Advanced Materials & Department of Chemistry, School of Chemistry and Molecular Engineering, East China University of Science and Technology, 130 Meilong Road, Shanghai, P R China; 2 Zhongshan Hospital Fudan University, 180 Fenglin Road, Shanghai, P R China

## Abstract

**Background:**

Biosensors have attracted increasing attention as reliable analytical instruments in *in situ* monitoring of public health and environmental pollution. For enzyme-based biosensors, the stabilization of enzymatic activity on the biological recognition element is of great importance. It is generally acknowledged that an effective immobilization technique is a key step to achieve the construction quality of biosensors.

**Results:**

A novel disposable biosensor was constructed by immobilizing laccase (Lac) with silica spheres on the surface of multi-walled carbon nanotubes (MWCNTs)-doped screen-printed electrode (SPE). Then, it was characterized in morphology and electrochemical properties by scanning electron microscopy (SEM) and cyclic voltammetry (CV). The characterization results indicated that a high loading of Lac and a good electrocatalytic activity could be obtained, attributing to the porous structure, large specific area and good biocompatibility of silica spheres and MWCNTs. Furthermore, the electrochemical sensing properties of the constructed biosensor were investigated by choosing dopamine (DA) as the typical model of phenolic compounds. It was shown that the biosensor displays a good linearity in the range from 1.3 to 85.5 μM with a detection limit of 0.42 μM (S/N = 3), and the Michaelis-Menten constant (K_m_^app^) was calculated to be 3.78 μM.

**Conclusion:**

The immobilization of Lac was successfully achieved with silica spheres to construct a disposable biosensor on the MWCNTs-doped SPE (MWCNTs/SPE). This biosensor could determine DA based on a non-oxidative mechanism in a rapid, selective and sensitive way. Besides, the developed biosensor could retain high enzymatic activity and possess good stability without cross-linking reagents. The proposed immobilization approach and the constructed biosensor offer a great potential for the fabrication of the enzyme-based biosensors and the analysis of phenolic compounds.

## Background

Laccase (Lac) has been widely used to construct electrochemical biosensors for phenolic and their derivatives, because it can catalyze the oxidation of phenolic compounds accompanied by the reduction of oxygen [1,2]. The high stability and enzymatic activity of the bioelectrochemical interfaces play a crucial role in the construction of Lac-based biosensors. The immobilization of enzymes on solid supports is one of the effective strategies, which allows the recovering and reusing of enzyme for several reaction cycles [3,4].

There is of intense interest in the construction of Lac-based biosensors using nanomaterials, due to their unique and particular properties [5]. Silica materials, which can accommodate different dimensions of enzyme without affecting their biological activity, could be considered as suitable hosts for enzyme immobilization [6,7]. For example, functionalized SBA-15 mesoporous silica was applied to immobilize Lac for the oxidation of a mixture of four phenolic compounds [8]. In another work, Lac was encapsulated into thin silicate film deposited on the Au electrode [9]. Moreover, magnetic mesoporous silica spheres were prepared to immobilize Lac as a promising support [10].

Multi-walled carbon nanotubes (MWCNTs), with high surface area and excellent biocompatibility, are also a promising candidate as the matrix material to incorporate enzyme and construct enzyme-based biosensors [11,12]. Importantly, because of the low overvoltage and rapid electrode kinetics, MWCNTs have the ability to facilitate electron transfer of enzyme with the electrode [13]. Therefore, MWCNTs have been employed as the supporting materials of Lac, such as the matrix based on MWCNTs-chitosan composite film [2], polyazetidine prepolymer-MWCNTs integrated system [14], and copper nanoparticles/chitosan/carboxylated MWCNT/polyaniline composite [15]. Recently, researchers are committed to develop MWCNTs/silica nanocomposite as the immobilization materials of Lac, because they possess excellent properties of low toxicity and good electrocatalytic activity, and could provide a stabilizing microenvironment for Lac [16-18].

Screen-printed electrode (SPE) is a kind of planar sensor device with various substrates that are coated with layers of electroconductive and insulating inks at controlled thickness [19]. Several works related to the biosensors construction have been reported based on the immobilization of Lac on the SPEs, which could be incorporated in portable systems as an alternative detection method for the direct *in-situ* analysis [20-22]. It is notable that the most common way to construct electrochemical interfaces is to drop conductive substrates onto the electrode surfaces [23,24]. However, it is difficult to produce thin (<1 mm) layers and control the consistency of detection [25]. While printing technology provides a convenient route to produce electrochemical sensors with consistent chemical performances based on the modification of functional conductive materials, such as conducting polymers [19], ionic liquid [25] and enzyme [26,27]-doped conductive materials.

**Figure 1:**
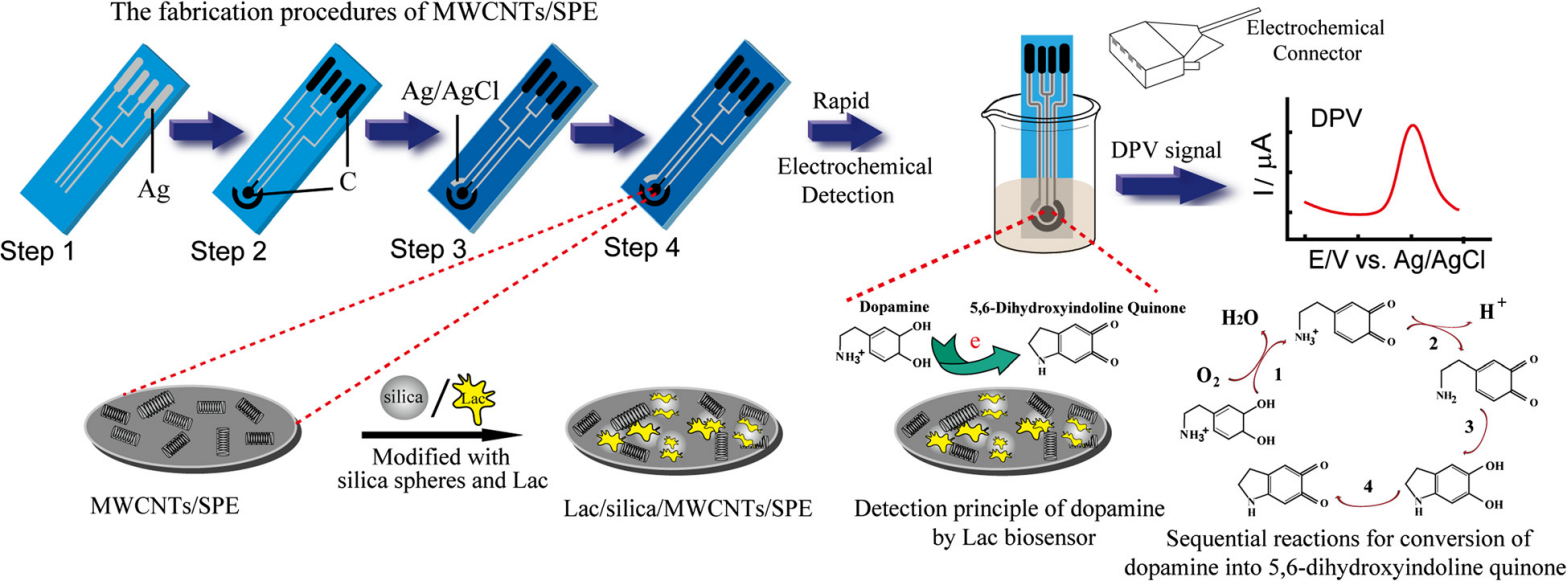
Schematic illustration of the screen-printed configuration, the procedures used in the process of MWCNTs-doped SPE fabrication (step 1 ~ step 4), and the detection procedures with the mechanism for the determination of DA at the disposable Lac/Si/MWCNTs/SPE (reaction 1 ~ reaction 4).

Herein, our goal is to construct a disposable electrochemical biosensor by immobilizing Lac on the MWCNTs-doped SPE using silica spheres as immobilization matrix (Lac/Si/MWCNTs/SPE) without cross-linking reagents. The morphology and the electrochemical properties of the constructed biosensor were characterized. Moreover, its electrochemical sensing properties were evaluated by selective measurements of dopamine (DA). Figure 1 depicts the procedures used for constructing the disposable biosensor and the mechanism for the determination of DA.

### Experimental

#### Reagents and apparatus

Laccase from *Trametes versicolor* (EC 1.10.3.2, 21.8 U mg^-1^), dopamine (DA), ascorbic acid (AA) and tetraethyl orthosilicate (TEOS) were purchased from Sigma-Aldrich (St. Louis, MO, USA). All other reagents were of analytical reagent grade purity and all solutions were prepared using deionized water obtained with a Mili-Q system (Millipore Co., Bedford, MA, USA). MWCNTs were supplied by Shenzhen Nanotech Port Co. Ltd. (Shenzhen, China) with a typical diameter of 10–30 nm and length of 5–15 μm, and their purity was 95-98%. Besides, silver paste, carbon paste, silver/silver chloride paste (Camnano Technology Ltd., Xuzhou, China), and insulating paste (Jujo Chemical Co., Ltd., Japan) were used to fabricate SPEs.

Scanning electron microscopy (SEM) results were obtained by using a Zeiss utra 55 field-emission SEM instrument (Zeiss, Germany). All the electrochemical measurements were performed with a CHI-1211A portable electrochemical workstation (Chenhua Instruments Co. Ltd., Shanghai, China). The measurements were performed at room temperature (~15°C).

#### Fabrication of MWCNTs/SPE

As the base electrodes for the printing process, SPEs with a standard three-electrode system and a 3.1 mm^2^ working area for each were fabricated according to the process described by our previous work [28,29] with an AT-25P screen-printing machine (ATMA CHAMPENT. Corp., China). Compared to our previous publications, the working electrodes were printed using different mass proportions of MWCNT/carbon paste, drying at 100°C. The prepared MWCNTs/SPEs were then stored at 4°C until required.

#### Construction of the disposable biosensor

Silica spheres were synthesized according to the Stöber’s method [30]. Typically, a solution of 5 mL of 33% ammonia solution was mixed with 50 mL of dry ethanol. After 3.14 mL of TEOS and 1.8 g of Milli-Q water was added in sequence, the solution was stirred to hydrolyze TEOS. After 12 h of stirring, a colloidal solution of silica spheres about 100 nm in diameter were obtained.

Before modification, the bare SPEs were pretreated in pH 7.0 potassium phosphate buffer solution (PBS) by applying an anodic potential of 2.00 V for 300 s. The synthesized silica spheres colloidal suspension was mixed with Lac (10.0 mg mL^-1^, prepared in 0.10 M pH 5.0 PBS) stock solution thoroughly in a volume ratio 2:3 for 24 hours. Then, 2.5 μL mixed solution of silica-Lac was coated onto the surface of the MWCNTs/SPE to form Lac/Si/MWCNTs/SPE as the disposable biosensor. After the solvent evaporated, the constructed biosensor was washed with deionized water to remove excess Lac. For comparison, Lac modified SPE (Lac/SPE), Lac modified MWCNTs/SPE (Lac/MWCNTs/SPE), and silica spheres modified MWCNTs/SPE (Si/MWCNTs/SPE) were fabricated with the similar steps. All of the modified electrodes were stored at 4°C.

## Results and discussion

### Morphology characterization of the disposable biosensor

**Figure 2:**
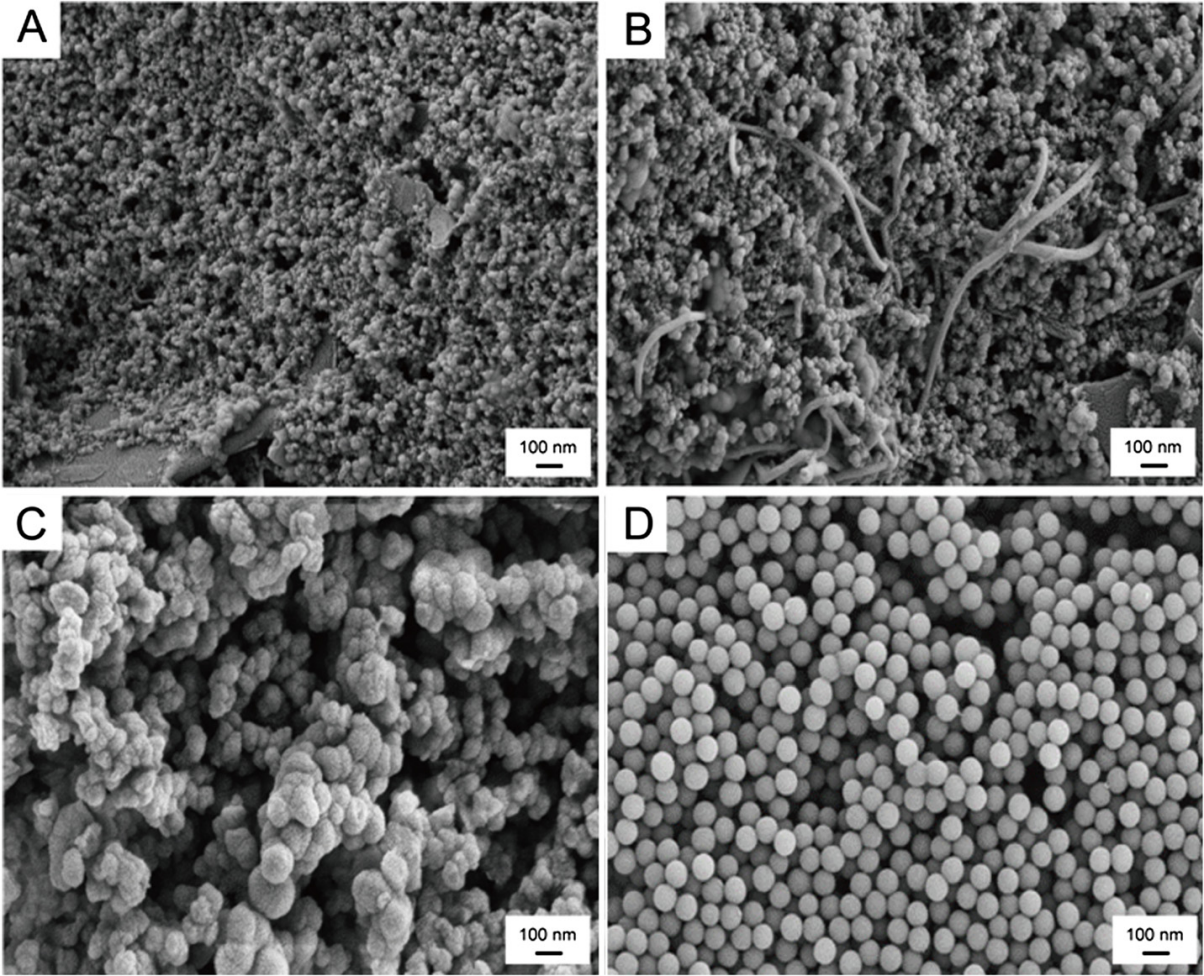
SEM images of (A) bare SPE, (B) MWCNTs/SPE, (C) Lac/Si/MWCNTs/SPE, and (D) silica spheres.

The typical SEM images of the disposable biosensor at different preparation stages are displayed in Figure 2. It can be seen that the surface of the bare SPE is covered by a layer of carbon particles (Figure 2A). On the contrary, on the MWCNTs/SPE, twisted MWCNTs distribute among the carbon particles to form a three-dimensional structure (Figure 2B). While, some of them are flat and embedded into the carbon particles, which may be due to the pressure during the screening process. After casting the silica solution loaded with Lac onto the MWCNTs/SPE, silica spheres can be found embedding into the Lac and connecting with each other (Figure 2C), which presented that Lac has been immobilized onto silica spheres successfully. For comparison, the SEM image of silica spheres is presented in Figure 2D.

### Electrochemical properties of the disposable biosensor

**Figure 3:**
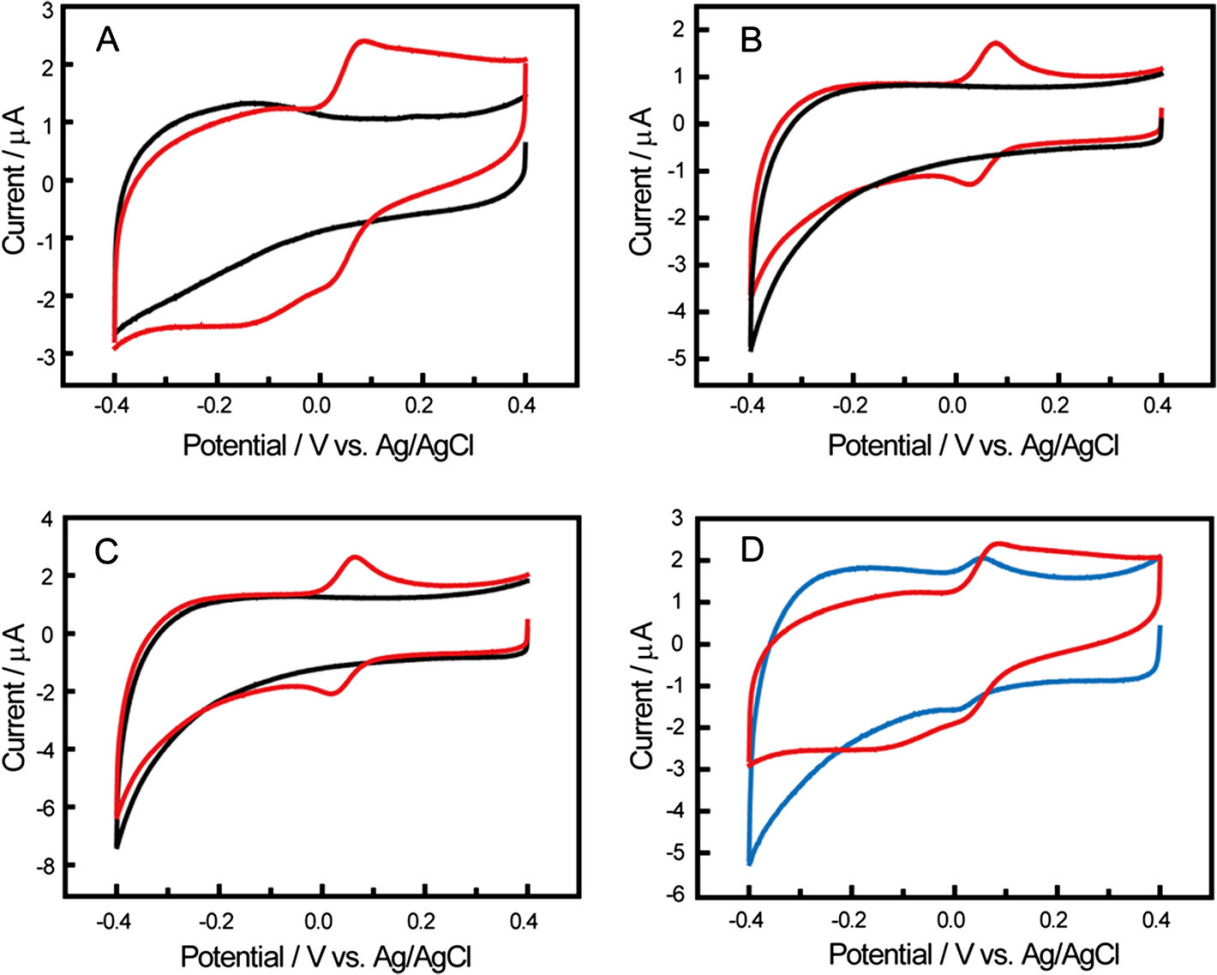
**CVs of Lac/Si/MWCNTs/SPE (A), Lac/SPE (B), Lac/MWCNTs/SPE (C) in 0.10 M PBS (pH 5.0) in the absence (black curves) and presence of 60 μM DA (red curves).** (**D**) CVs of Si/MWCNTs/SPE (blue curve) and Lac/Si/MWCNTs/SPE (red curve) in 0.10 M PBS (pH 5.0) in the presence of 60 μM DA. Scan rate: 50 mV s^-1^.

The electrochemical sensing properties of the disposable biosensor were investigated by choosing DA as the typical model of phenolic compounds. In Figure 3, the electrocatalytic properties of the Lac/Si/MWCNTs/SPE (Figure 3A), Lac/SPE (Figure 3B), Lac/MWCNTs/SPE (Figure 3C), and Si/MWCNTs/SPE (Figure 3D) for DA were compared by performing cyclic voltammetry (CV) experiments in PBS solution (pH 5.0). As we expected, no catalytic current responses are shown in the absence of DA (black curves in Figure 3A, B and C). In contrast, upon the presence of DA in solution, a pair of well-defined redox peaks is obtained (red curves in Figure 3B and C, and blue curve in Figure 3D). However, the voltammetric feature of the Lac/Si/MWCNTs/SPE (red curve in Figure 3A) differs significantly, because a cathodic peak at around −0.158 V vs. Ag/AgCl appears. The height of this cathodic peak is sensitive to the change of the DA concentration in PBS (not shown here). The possible reason is essentially based on the demonstrated non-oxidative electrochemical approach [31] by taking advantage of the chemical properties of DA and the catalytic activity of Lac, as shown in Figure 1 (reaction 1–4). DA can be oxidized into its quinonoid form (Figure 1, reaction 1) either through a reversible electrochemical method or an irreversible chemical method under the catalysis of Lac, which can be described simply as follows [7]:

(1) $$\begin{array}{l} o,m,p-\text{benzenediol}+1/2\;{\text{O}}_{2}\overset{\text{laccase}}{\to }o,m,p\\ \;-\text{quinone}+{\text{H}}_{2}\text{O} \end{array}$$

(2) $$\begin{array}{l} o,m,p-\text{quinone}+{\text{H}}^{+}+2{\text{e}}^{-}\\ \;\to o,m,p-\text{benzenediol} \end{array}$$

The quinones formed in reaction (1) are usually electrochemically active and subsequently re-reduced on the surface of the electrode at the appropriate potentials. DA, as a typical model of *o*-benzenediol, follows this reaction mechanism. Then, Lac is used to initialize the sequential intramolecular cyclization reactions of DA, including a deprotonation reaction (Figure 1, reaction 2), an intramolecular cyclization process (reaction 3), and a disproportionation reaction and/or oxidation (reaction 4). The finally formed 5,6-dihydroxyindoline quinone is readily electrochemically reduced at SPE [32]. On the basis of these reaction properties of DA, the non-oxidative electrochemical approach can be proposed for the determination of DA by measuring the cathodic current of 5,6-dihydroxyindoline quinone at a negative potential (−0.158 V).

The results of Figure 3B and C show that symmetrical redox couple of DA at the Lac/SPE and the Lac/MWCNTs/SPE with the potential difference between anodic and cathodic peaks (ΔE_p_) are 0.051 V and 0.044 V, characteristic of a two-electron and two-proton quasi-reversible redox process of DA at both SPEs [32]. No cathodic peak at around −0.150 V is found at these SPEs. The results demonstrate that the Lac/SPE or the Lac/MWCNTs/SPE does not have any appreciable electrocatalytic activity to DA based on a non-oxidative electrochemical approach, implying the direct immobilization of Lac on bare SPE or MWCNTs/SPE is not successful. Moreover, the electrocatalytic features of the Si/MWCNTs/SPE to DA are similar to those of the Lac/SPE and the Lac/MWCNTs/SPE in terms of the anodic (0.057 V) and cathodic (0.015 V) peak potentials (blue curve in Figure 3D), and there is still no cathodic peak at around −0.150 V appearing at this Si/MWCNTs/SPE. However, after immobilizing Lac on the surface of the MWCNTs/SPE with silica spheres, the cathodic peak caused by the enzymatic oxidation of DA appears at around −0.158 V (red curve in Figure 3D). Obviously, this process is ascribed to the two-electron and two-proton quasi-reversible redox process of 5,6-dihydroxyindoline quinone, and implies that the Lac has been immobilized on the Lac/Si/MWCNTs/SPE stably with a good biocatalytic activity [2]. Furthermore, the introduction of Lac makes the measurement of DA through cathodic current at negative potential (around −0.150 V) achieve, which avoiding the interference of other electroactive species whose oxidized potentials are very close to DA, by measuring the oxidation current of DA at a positive potential. These phenomena substantially demonstrate that the disposable biosensor can show an excellent electrocatalytic activity to DA based on this non-oxidative electrochemical approach. On one hand, the biosensor could retain the bioactivity of Lac to a large extent by immobilization of Lac with silica spheres on the MWCNTs/SPE. On the other hand, silica spheres and MWCNTs can both provide large loading area for Lac by their high specific surface area. The above results also imply that the Lac immobilized on the surface of Si/MWCNTs/SPE might provoke the drastic conformation change of Lac which is in favor of the active sites of enzyme approaching the SPE. However, if cross-linking reagents were used to immobilize Lac, this maybe promotes a high degree of reticulation with Lac that blocks the process [2].

### The effect of pH on the electrochemical properties of the disposable biosensor

**Figure 4:**
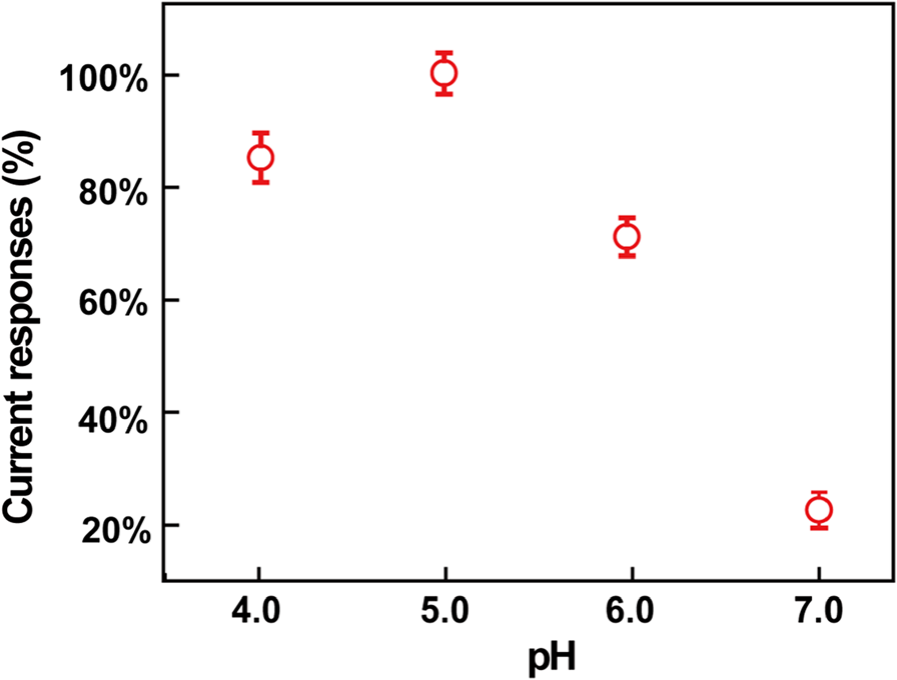
**Relative current responses of the Lac/Si/MWCNTs/SPE at different pH values, containing of 60 μM DA.** The cathodic currents are expressed as the percentage of the maximum response obtained at pH = 5.0.

Since the proton participates in the electrochemical reaction, the pH value of the supporting electrolyte is considered to be an important parameter affecting the electrochemical behavior of the biosensor [28]. The current responses to DA of the disposable biosensor in the pH range from 4.0 to 7.0 were evaluated. The results are shown in Figure 4, where the cathodic currents of DA at the Lac/Si/MWCNTs/SPE are expressed as the percentage of the maximum response obtained at an appropriate pH. It is shown that the optimum response is obtained at about pH 5.0, just below the neutral pH, which is consistent with the reported work [7]. It is noteworthy that although the soluble Lac has an optimum pH value at around 3.0-4.0 to retain its bioactivity [33], the Lac immobilized with silica spheres on MWCNTs/SPE makes the effective pH values shift to 4.0-6.0. This advantage renders the Lac/Si/MWCNTs/SPE for broad application fields. Therefore, a pH value of 5.0 was selected for next experiments.

### The effect of the amount of MWCNTs on the electrochemical properties of the disposable biosensor

**Figure 5:**
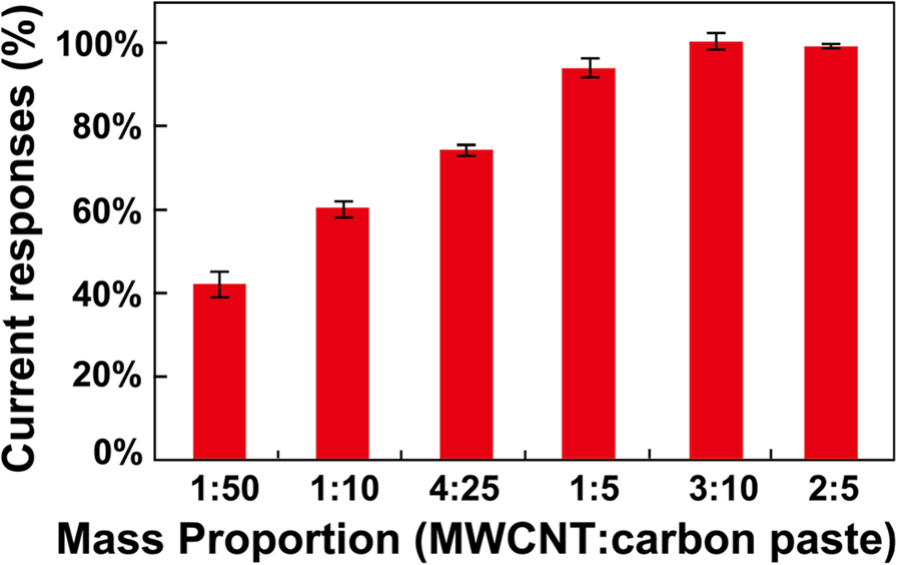
**Relative current responses of the Lac/Si/MWCNTs/SPE loading variable amount of MWCNTs to 60 μM DA in 0.10 M PBS (pH 5.0).** Scan rate: 50 mV s^-1^. The cathodic currents are expressed as the percentage of the maximum response obtained at mass proportion of MWCNTs/carbon paste of 3:10. The mass proportion of MWCNTs/carbon paste (MWCNTs:carbon paste) in the process of screen-printing is 1:50, 1:10, 4:25, 1:5, 3:10, and 2:5, respectively.

Another important parameter affecting the responses of the target analyte at the disposable biosensor is the loading amount of MWCNTs. SPEs doped with different mass proportions of carbon paste and MWCNTs were investigated in order to choose an optimum loading amount of MWCNTs. As shown in Figure 5, with the mass proportion of MWCNTs/carbon paste (MWCNTs: carbon paste) changing from 1:50 to 2:5, the cathodic current response (expressed as the percentage of the maximum response) of DA at the Lac/Si/MWCNTs/SPE enhances, and reaching its maximum at 3:10. Therefore, the proportion of 3:10 was chosen for the fabrication of subsequent disposable Lac/Si/MWCNTs/SPEs.

### Determination of DA using the disposable biosensor

**Figure 6:**
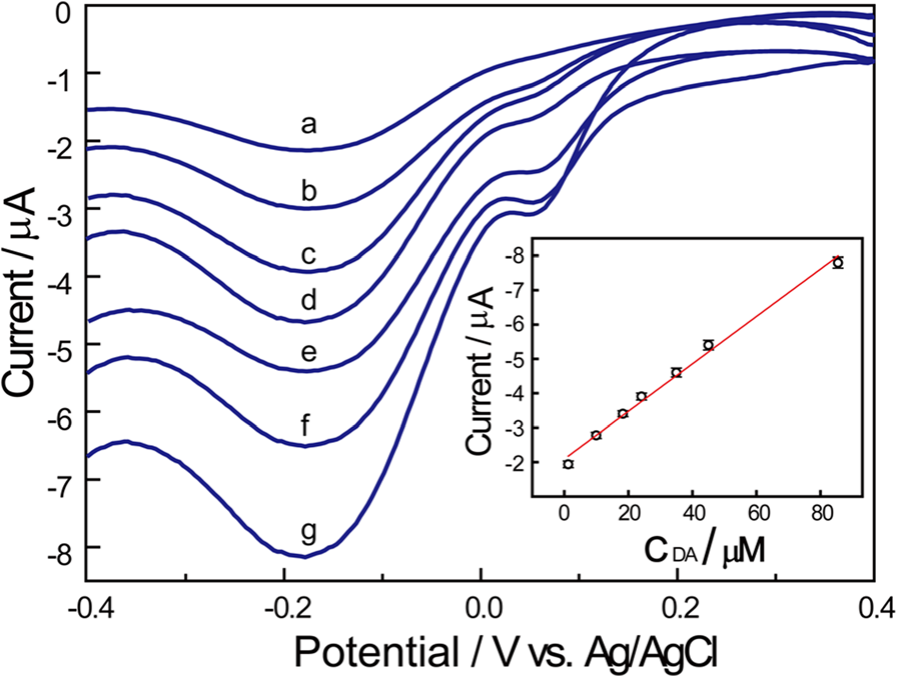
**DPVs obtained at the Lac/Si/MWCNTs/SPE in 0.10 M PBS (pH 5.0) containing (a) 1.3 μM, (b) 10.0 μM, (c) 18.3 μM, (d) 24.1 μM, (e) 35.0 μM, (f) 45.0 μM, (g) 85.5 μM.** Inset: the plot of reduction current responses against the concentrations of DA.

The usage of Lac enables DA determination with differential pulse voltammetry (DPV), which shows a better resolution and a higher signal-to-noise ratio comparing with CV [31]. As displayed in Figure 6, the typical DPVs at the Lac/Si/MWCNTs/SPE with different concentrations of DA are obtained. It can be observed that the cathodic peak current recorded at around −0.177 V enhances with the increasing of the concentration of DA in solution, and the current is found to be linear with the concentration of DA from 1.3 to 85.5 μM (I (μA) = −0.069C_DA_ (μM) – 2.091, R = 0.9908) (Inset in Figure 6). The detection limit was calculated to be 0.42 μM (S/N = 3). Furthermore, the relative standard deviation (R.S.D.), sensitivity, and Michaelis-Menten constant (K_m_^app^) of the disposable biosensor were evaluated (Table 1). Among them, the K_m_^app^ value, which can provide information regarding the Lac-substrate kinetics, is calculated according to the Michaelis-Menten equation [34]:

$$I_{\text{max}}/I_{\text{s}}={K_{\text{m}}}^{\text{app}}/\text{C}+1$$

where I_s_ refers to the steady-state catalytic current, I_max_ is the maximum current measured under saturated conditions, and C is the concentration of DA. The K_m_^app^ value was estimated to be 3.78 μM. This small K_m_^app^ means that Lac immobilized with silica spheres on the MWCNTs/SPE possesses very high enzymatic activity for the determination of DA. These results indicate that the disposable biosensor has a good analytical performance for DA.

**Table 1:** The response properties of the disposable biosensor to DA

**Linear range (μM)**	**Correlation coefficient**	**Detection limit (μM)**	**R.S.D (n=5)**	**Sensitivity (μA mM**^**-1**^ **cm**^**-2**^**)**	**K**_**M**_^**app**^**(μM)**
1.3 to 85.5	0.9908	0.42	4.0%	2.787 × 10^3^	3.78

**Figure 7:**
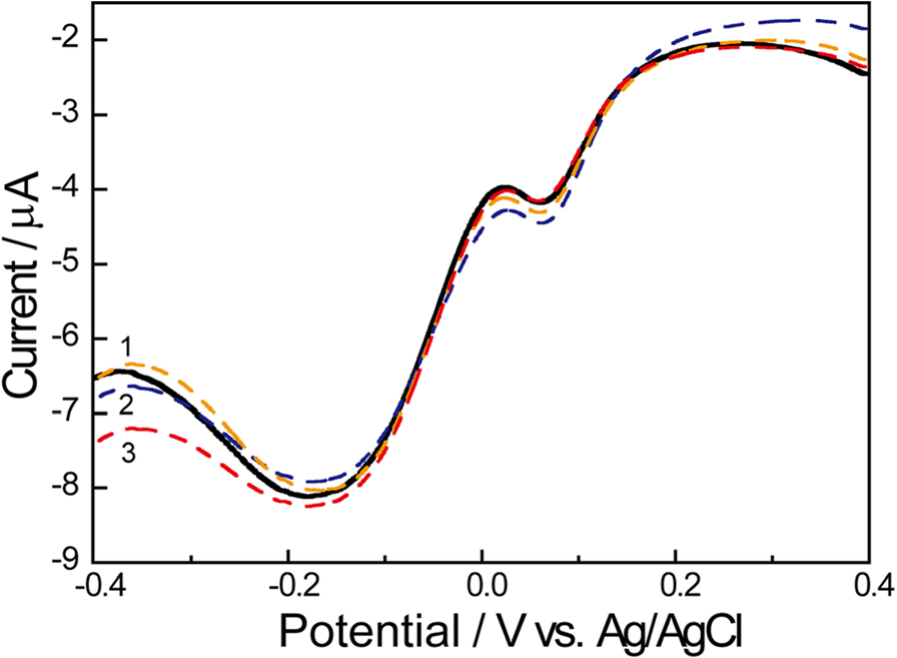
**The selective determination of DA in the coexistence of AA.** The solid curve stands for DPV obtained in 0.10 M PBS (pH 5.0) containing 85.5 μM DA. The dash curves 1, 2 and 3 represent DPVs obtained after introducing 20.0 μM (curve 1), 40.0 μM (curve 2) and 60.0 μM (curve 3) AA, respectively.

The selectivity of the disposable biosensor to DA determination was also investigated. AA was added into the solution which contained 85.5 μM DA (dash curves 1–3 in Figure 7). As can be seen from Figure 7, the introduction of AA into DA solution does not lead to an obvious change in the cathodic peak current responses, indicating that DA can be virtually detected using Lac/Si/MWCNTs/SPE due to the interference-free from AA. These results may suggest the disposable biosensor could be used for the practical measurements of DA based on the non-oxidative mechanism.

### Stability, reproducibility and repeatability of the disposable biosensor

One of the most critical issues in constructing biosensor is the avoidance of enzyme immobilized on the surface of the electrode leaking into the solution. In this study, the stability of Lac/Si/MWCNTs/SPE was investigated by recording cathodic peak current responses of the Lac/Si/MWCNTs/SPE at different sweep segments in 0.10 M PBS (pH 5.0) containing of 60.0 μM DA. It was found that the current responses stayed at the same level after 20 sweep segments, indicating that Lac was immobilized stably on the SPE. The results further proved that the proposed immobilization method was effective and it was not necessary to use any other cross-linking reagents. The possible reason may be due to the large loading area, good biocompatibility and the stabilizing property provided by silica spheres and MWCNTs, attributing to their porous and three-dimensional architecture. The nanosized pores on silica spheres and MWCNTs could act as small cages surrounding the Lac, consequently offering a protective chemical microenvironment which is similar to the microenvironment near enzyme in biological cells. Furthermore, the interconnected pores and a well-defined three-dimensional network of the proposed immobilization matrix can prevent Lac from leaching into the solution while allow free diffusion between the matrix and product molecules from/to the catalytic active sites. Therefore, even if in the absence of cross-linking reagents, the developed biosensor can still show good stability during the detection [35,36].

To verify the reproducibility of the disposable biosensor, five different Lac/Si/MWCNTs/SPEs fabricated by same steps independently were chosen randomly from 50 pieces of store SPEs. The R.S.D. for the cathodic peak current responses to 60.0 μM DA was 6.5%, meaning that the constructing procedures were reliable and the modified SPEs had a good reproducibility. In addition, the same Lac/Si/MWCNTs/SPE was used to detect DA for five times successively. As a result, the R.S.D. value was 4.7%, showing a good repeatability.

The storage stability of the disposable biosensor was also investigated. After 10 and 30 days saving at 4°C, the current response of Lac/Si/MWCNTs/SPE reached to 91.0% and 86.0% of the initial response respectively in the 60.0 μM DA solution. The good stability may ascribe to the effective protection of the bioactivity of Lac due to the consistent stability of silica spheres and the biocompatible microenvironment provided by silica spheres and MWCNTs.

## Conclusions

A novel disposable biosensor has been successfully constructed on MWCNTs/SPE by immobilizing Lac with silica spheres. Due to the large specific surface area and excellent biocompatibility of MWCNTs and silica spheres, the biosensor can effectively provide a suitable microenvironment for the immobilization of Lac and exhibit a good electrocatalytic performance for DA. In addition, based on a non-oxidative electrochemical mechanism, the biosensor enables the *in situ* determination of DA with good sensitivity, selectivity and reproducibility. In summary, the proposed approach of enzyme immobilization shows a great potential for the construction of biosensors without using cross-linking reagents, and the constructed biosensor displays an excellent analytical performance for phenolic compounds in a rapid and cost-effective way.

## Competing interests

The authors declare that they have no competing interests.

## Authors’ contributions

YL carried out the experimental work, participated in data collection and analysis, involved in drafting and revising the manuscript. ML participated in fabricating SPEs and data analysis. LZ, DL, ZP revised it critically for important intellectual content, and gave final approval of the version to be published. All authors read and approved the final manuscript.
